# The Potential Role of Integrin Signaling in Memory and Cognitive Impairment

**DOI:** 10.3390/biom13010108

**Published:** 2023-01-05

**Authors:** Ifechukwude Joachim Biose, Saifudeen Ismael, Blake Ouvrier, Amanda Louise White, Gregory Jaye Bix

**Affiliations:** 1 Clinical Neuroscience Research Center, Department of Neurosurgery, Tulane University School of Medicine, New Orleans, LA 70112, USA; 2 Tulane Brain Institute, Tulane University, New Orleans, LA 70112, USA; 3 School of Medicine, Tulane University, New Orleans, LA 70112, USA; 4 Department of Neurology, Tulane University School of Medicine, New Orleans, LA 70112, USA; 5 Department of Microbiology and Immunology, Tulane University School of Medicine, New Orleans, LA 70112, USA; 6 School of Public Health and Tropical Medicine, Tulane University, New Orleans, LA 70122, USA

**Keywords:** integrins, extracellular matrix, dementia, and blood–brain barrier

## Abstract

Dementia currently has no cure and, due to the increased prevalence and associated economic and personal burden of this condition, current research efforts for the development of potential therapies have intensified. Recently, targeting integrins as a strategy to ameliorate dementia and other forms of cognitive impairment has begun to gain traction. Integrins are major bidirectional signaling receptors in mammalian cells, mediating various physiological processes such as cell–cell interaction and cell adhesion, and are also known to bind to the extracellular matrix. In particular, integrins play a critical role in the synaptic transmission of signals, hence their potential contribution to memory formation and significance in cognitive impairment. In this review, we describe the physiological roles that integrins play in the blood–brain barrier (BBB) and in the formation of memories. We also provide a clear overview of how integrins are implicated in BBB disruption following cerebral pathology. Given that vascular contributions to cognitive impairment and dementia and Alzheimer’s’ disease are prominent forms of dementia that involve BBB disruption, as well as chronic inflammation, we present current approaches shown to improve dementia-like conditions with integrins as a central focus. We conclude that integrins are vital in memory formation and that their disruption could lead to various forms of cognitive impairment. While further research to understand the relationships between integrins and memory is needed, we propose that the translational relevance of research efforts in this area could be improved through the use of appropriately aged, comorbid, male and female animals.

## 1. Introduction and Integrin Signaling Mechanics

Integrins are major receptors in mammalian cells, mediating various physiological processes such as cell–cell interaction and cell adhesion, and are also known to bind to the extracellular matrix (ECM). Recently, the interaction between integrins and the ECM has been ascribed to information processing and memory functions via its mechanosensory functions and maintenance of dendritic integrity at the synapse [1]. Whilst the role of integrins in cognitive impairment has been understudied, the main role of integrin interactions with the ECM allows them to function as signal transducers in synergy with other classes of receptors in the activation of intracellular processes to promote cell proliferation, cell differentiation, cell survival, and cell growth [2]. Understanding the mechanics of integrin functions is vital to determining molecular targets for potential therapeutic applications. To facilitate this, here we briefly describe the role and interactions of the ECM with integrins. Further, we describe integrins’ role in blood–brain barrier (BBB) integrity and memory formation, as well as their potential therapeutic applications in ameliorating dementia.

Most cells are anchored in the ECM, a complex of various proteins such as collagen IV, laminin, and perlecan, which allows for sufficient tensile and adhesive strength to support and maintain the structure and orientation of cells. The ECM conveys many benefits to the surrounding cells, such as allowing for mechanical, biochemical, and electrical forces to impose changes on the cells in different and highly specific ways [3]. Cell adhesion to the ECM, vital for functional multicellularity, is accomplished by integrins, which are bidirectional transmembrane receptors that allow for both mechanical and biochemical signaling between cells and the ECM [3,4]. Connecting within cells to the intracellular actin cytoskeleton, integrins allow for the mechano-transduction of signals to induce functional and conformational changes in both the ECM proteins and cellular components. Some of these signaling events that modulate cells are proliferation, shape, polarity, motility, gene expression, and differentiation [5]. Additionally, the protective effects of integrins are directly related to essential physiological processes such as cell survival and proliferation: to block apoptosis, via phosphatidylinositol 3-kinase and protein kinase B (also known as Akt; i.e., PI3K-Akt) signaling, and to stimulate cell cycle progression, via extracellular-signal-regulated kinase (ERK) and cyclin D1 signaling [4,5].

All these cellular conformational and functional changes hinge upon integrin binding and signaling. The integrin family is composed of 18 alpha and 8 beta subunits, which form 24 distinct integrin heterodimers. Integrins mediate two different kinds of signaling pathways: “inside-out” and “outside-in”. The “inside-out” pathway occurs when an intracellular signal promotes the binding of specific proteins, which may induce conformational changes on the integrin and increase the affinity for ECM ligands. However, the “outside-in” pathway recruits protein complexes that regulate cell function such as proliferation and differentiation [4]. These “inside-out” and “outside-in” signaling roles of integrins are accomplished via mechano-transduction between the ECM and intracellular actin cytoskeleton [4]. Mechanical loads on tissue are perceived by cells as stimuli via the surrounding ECM through integrin signaling. The perception of these stimuli by cells is critical for the cell–matrix interactions that regulate the mechanical homeostasis of tissues [3,6]. Integrins also have some association with memory formation due to their abundant expression at the synapses as well as their unique facilitation of mechanical homeostasis.

### Integrins and the Blood–Brain Barrier

In the absence of a constant mechanical stimuli, integrins can induce structural apoptosis in the surrounding parenchyma, which may increase vascular permeability and thereby weaken the integrity of the BBB [6,7]. Hence, integrins reserve an integral physiological function in the maintenance of the BBB and cerebral homeostasis. Integrins can potentially be a therapeutic target to maintain BBB integrity in the event of cerebral pathology.

Integrin signaling is also involved in the promotion and maintenance of the selective permeability of the BBB [8,9]. Many integrin knock-out (KO) mouse models are lethal or develop brain defects. For example, β1 KO mice have decreased BBB integrity [8,10]. Similarly, α5 KO mice show increased BBB leakage [9]. Understanding the specific role of each of the outlined integrin, not only with regard to their role in inflammation but specifically for their unique contribution to the BBB, is germane to maintaining a healthy brain as well as developing potential therapies.

A common insult to BBB integrity is cerebral ischemia and/or vascular-dysfunction-induced oligemia. Cerebral ischemia/oligemia occurs when blood flow to downstream microvasculature or brain parenchyma is impaired, generating a core of dead brain cells with a salvageable peri-infarct region, called the penumbra. The integrity of the BBB in the penumbral region is subject to pro-apoptotic factors in a physiological and compensatory effort by the brain towards angiogenesis. Although various other neurovascular factors play a significant role in the maintenance of the BBB integrity, we [11] and others [12] demonstrated a reduced level of tight junction protein expressions for up to 14 days in the ischemic core. Similarly, in the peri-infarct region, it was reported that tight junction protein expression is also lowered immediately and in the first 4 days following cerebral ischemia induction; a slow restoration of normal tight junction protein expressions ensues thereafter [12]. These findings are in tandem with angiogenesis following ischemic stroke and were associated with increased endothelial α5β1 integrin expression [12]. The disruption of the BBB may have a direct effect on cognitive deficit and long-term functional recovery following brain ischemia.

Integrins, such as α5β1 and α2β1 receptors, contribute highly to angiogenesis and vascular remodeling and are under intense research focus as therapeutic targets. The α5β1 integrin is largely expressed in the endothelial cells of the cerebral vasculature. This is of importance since the c-terminal ligand of the perlecan domain V (DV, an ECM protein, which is cleaved by proteases and richly expressed in the brain following cerebral ischemia) binds with the α5β1 integrin receptor resulting in a pro-angiogenic effect through VEGF upregulation and the ERK signaling pathway [13]. DV is an 85kDa c-terminal domain of perlecan consisting of three laminin-like globular domains (LG1-LG3) and LG3 is the c-terminal domain mostly released by proteolytic cleavage [14]. It is well documented that the increased expression of α5β1 integrin is associated with a similar increased brain expression of angiopoietin-1 (Ang1) following ischemic stroke [12,15]. Ang1 is a vascular ligand for Tie2. Tie2 expression induces endothelial progenitor/cell migration and protects from apoptosis via the upregulation of focal adhesion kinase and Akt signaling, resulting in vascular protection, cell migration, and tube formation only when α5β1 integrin is upregulated [9,16]. However, Ang1 signaling and angiogenesis interferes with the β1 conformation of the ECM components, which impairs BBB integrity and results in increased BBB permeability [17]. Indeed, β1 integrins promote coagulation and phagocytosis, which are essential steps in angiogenesis, and this is likely responsible for the BBB permeability following brain ischemia [18].

While we have shown that selectively inhibiting the α5β1 integrin receptor with the small peptide ATN-161 in acute ischemic stroke ameliorates BBB disruption [11], we reason that the tandem increase in Ang1 and α5 integrin expression beyond the acute phase of cerebral ischemia may help stabilize the BBB and improve local blood flow through promoted angiogenesis as shown by other groups [12,15]. Consequently, inhibiting α5β1 integrin during the acute phase of ischemic stroke, but potentially not in the long term, could help stabilize the BBB and ameliorate neuroinflammatory processes, which holds the key to post-stroke outcomes.

Since the modulation of integrins results in changes in the brain, there is sufficient reason to believe that integrins play a role in memory pathology, such as dementia, where BBB disruption is a commonality. Below we describe a more direct involvement of integrins in memory formation.

## 2. Integrins and Memory

**Figure 1:**
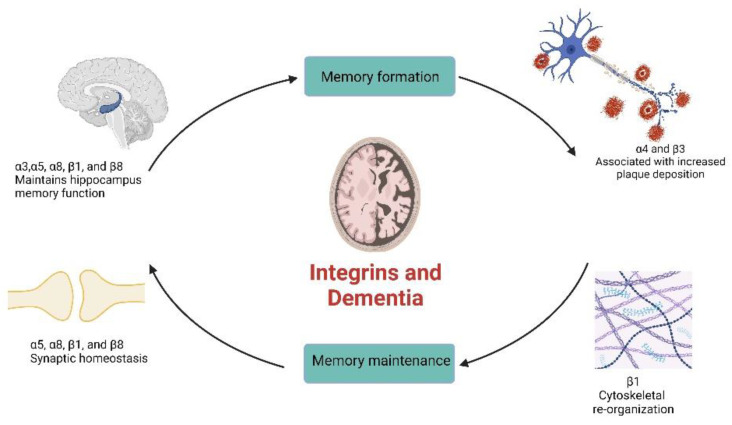
The potential role of integrins in memory formation and maintenance. Various integrins play vital roles in maintaining the number and size of the dendritic spine at the synapse as well as in functional hippocampal memory formation and retention. In addition, β1 integrins are responsible for the re-assembly of the cytoskeleton following synaptic transmission of signals. Other integrins are activated and associated with plaque deposition in human brain samples obtained from individuals with dementia-like symptoms. The inhibition/deletion of these integrins revealed functional loss of memory formation/retention; hence, targeting these integrins will be critical in illuminating their potential therapeutic values.

The role of integrins in memory function and the formation of new memories lies at the synaptic connection. Memory is stored and processed from the pattern of molecular changes involved at the presynaptic and post-synaptic terminals during the transmission of neuronal signals. The unique biochemical conformation and interaction of the ECM, cytoskeletal structures, and integrins allow neurotransmitter release and trafficking, which, along with other biochemical processes, enable signal transduction and processing. Ultimately, synaptic signal transmission is facilitated by the adhesive and signaling functions of integrins. Long-term potentiation (LTP), which describes the extended increased synaptic transmission of signals between two or more neurons based on prior persistent patterns of biochemical processes at the synapse, has been attenuated in Drosophila by integrin inhibitors or other pharmacological compounds [19,20,21,22]. Similarly, disruption of the integrin-associated protein (IAP, also known as CD47), causes memory impairment in mice due to its relationship with one of the genes related to memory formation. Moreover, the inhibition of IAP in the dentate gyrus of rat hippocampus impairs both synaptic plasticity and behavioral plasticity resulting in reduced memory retention and LTP [23,24,25]. Since learning and memory are a result of the constant alterations at the synaptic connections, the interplay between integrins and the ECM may be directly linked to memory formation. Hence, pathological or abnormal changes in the conformation of integrins may result in lapses in memory formation and retention (Figure 1). The first direct evidence for the role of integrins in memory function emanated from the study of Chan et al. [26]. They found that the concurrent attenuated expression of α3, α5, and α8 integrin subunits resulted in spatial memory deficit during Morris water maze (MWM) tests. This early finding showed that integrin receptors, which are known to play a role in cell adhesion, may also mediate behavioral plasticity. Further, the same study and other reports demonstrated that the specific deletion of α3 or β1 integrins in the forebrain and excitatory neurons impairs working memory in the hippocampal-dependent test of T-maze [26,27,28]. Additionally, α8 and β8 integrins are abundant at the dendritic spines of pyramidal neurons and associated with post-synaptic density [29,30]. α5 integrins are also richly localized in the apical dendrites of the pyramidal cells of the cortex and hippocampus [31]. This suggests that integrins are actively involved in the processing of memory function.

Using rodent hippocampal slices, several studies demonstrated the direct dependence of LTP on integrins. LTP was attenuated when α3 and α5 integrins were inhibited with antibodies from snake venoms [22,31]. Moreover, both LTP and the biochemical restoration of actin assembly were eliminated when β1-integrin inhibition was induced immediately after stimulation [32]. Despite the mounting body of evidence linking integrins to memory functions, it is not known whether integrins directly regulate aspects of memory formation and recall while performing their intracellular signaling or cell adhesion roles. This level of understanding will provide essential evidence to further integrin targeting to potentially improve memory functions.

The β-1 integrin subunit is the most commonly occurring subunit; this includes α4β1, α5β1, α6β1, αVβ1, αVβ3 α2β1, and α11β1. The downstream signaling functions of β1 integrins occurs via interactions with non-receptor tyrosine kinase Arg to modulate dendritic and synaptic plasticity in the hippocampal neurons [33]. Arg binds to and phosphorylates the intracellular tail of the β1 integrin at the dendritic spines where Arg is richly expressed [34,35,36]. Consequently, when the β1 integrin is conditionally knocked out in mice, hippocampal-dependent memory deficits were observed due to a significant reduction in the size and quantity of the dendritic spines and synapses [33]. A similar observation was noted in mice with homozygous deletion of Arg [36,37]. Moreover, the direct inhibition of the β1 integrin attenuated the quantity of synapses in the apical dendrites of CA1 pyramidal neurons [38]. These suggest that β1 is crucial not only for the early formation of synapses but also for the maintenance of hippocampal memory functions. Similarly, the deletion of the α5 integrin in the hippocampal neurons results in a reduction in synapses as well as dendritic spines [39].

Early studies reported the association of integrins with age-associated memory deficits. Aged human hippocampal and cortical neurons were immunoreactive for the α4 integrin subunit, which was not observed in samples from young adults [40]. Moreover, tau-positive plaques in samples from patients who had Alzheimer’s disease (AD) reacted to antibodies for the α4 integrin subunit [40]. Relatedly, the senile plaques and neurofibrillary tangles in human brain samples from AD patients was highly reactive to antibodies for the β3 integrin [41]. This suggests that the increased deposition of plaques activated α4 and β3 integrins. The integrins’ specific role(s) in AD or other diseases that impact memory functions is not well understood and calls for further investigations.

However, it has been shown that increased integrin expression at the site of tau-positive plaque formation could be the brain’s attempt to rid itself of the plaques. Activated microglial cells in the region of amyloid plaques from the brain samples of dementia patients have a higher expression of α4β1 and αLβ2 integrins [42]. Experimental findings from rats demonstrate the colocalization of α1β1 and α5β1 integrins with β-amyloid precursor proteins in hippocampal neurons and cortical astrocytes [43,44]. Integrins are clearly implicated in the inflammatory response to abnormal brain processes that impair memory function. Hence, understanding the specific roles integrins play in cognitive dysfunction may be the first step towards the development of therapeutic strategies for dementia.

Given the importance and involvement of integrins in proper brain health and development, integrins could be a potential therapeutic target for dementia. In fact, coinciding with the growing aging population, incidences of dementia are also expected to increase and become an even greater healthcare burden [45,46]. In 2010, healthcare for dementia-related cases in the United States cost over 100 billion USD, and costs are predicted to double to over 250 billion USD by the year 2040 [47]. Population studies estimate that around 50 million people worldwide have been diagnosed with dementia and that dementia cases are only going to increase, with some estimates saying cases will triple, i.e., 150 million, by 2050 [48]. Therefore, increased efforts towards understanding the potential therapeutic roles of integrins may help reduce the economic impact of dementia. Below, we describe the two forms of dementia, vascular contributions to cognitive impairment and dementia (VCID) and AD, which may be well suited for increased integrin research endeavors.

### 2.1. Vascular Contributions to Cognitive Impairment and Dementia (VCID)

VCID is a term used to describe any degree of cognitive impairment caused by cerebrovascular dysfunction. VCID encompasses patients suffering from vascular cognitive impairment to the more severe diagnosis of vascular dementia. VCID represents a growing major health concern worldwide. VCID alone is the second leading cause of dementia and accounts for 20% of all dementia cases in Europe and North America and 30% in Asia [49]. While aging is one of the main risk factors, smoking, inflammation, hypertension, and stroke have all been shown to increase the risk of developing VCID [45,50].

VCID is a complex disease that can be caused by many different factors. In general, an event causing blood flow dysregulation or hypoperfusion in the brain leads to a decrease in glucose metabolism, vascular permeability, and ultimately to neuronal death. The processes that follow cerebral blood flow insufficiency are neuroinflammation, vascular remodeling, and BBB disruption. Chronic hypoperfusion has been shown to have a strong relationship in VCID pathogenesis likely leading to pathology such as infarcts, hemorrhages, and memory impairment in rodents [45]. In addition, cerebral small vessel disease, which results from chronic hypertension and cerebrovascular remodeling, leads to cognitive impairment [51]. Consequently, an excellent animal model is the spontaneously hypertensive rat (SHR), which develops cerebral small vessel disease early in life, developing white matter damage and a dysfunction of the BBB in later life [51].

Since BBB integrity is highly implicated in the pathogenesis of VCID, it is imperative to increase research efforts in understanding how integrins can be targeted to maintain BBB integrity and potentially ameliorate memory function. Only one study from our group has explored the expressions of the α5 integrin following 14 days of bilateral carotid artery stenosis in mice, a valid model of VCID [52]. We found an increase in the cortical and striatal expressions of the α5 integrin, a decrease in tight junction proteins, and a substantial BBB permeability 14 days after bilateral carotid artery stenosis in young adult males. This is suggestive of active angiogenesis, the brain’s compensatory attempt to increase the number of blood vessels, which ultimately disrupts optimal BBB function and may potentially lead to cognitive impairments.

### 2.2. Alzheimer’s Disease (AD)

In patients with AD, several integrins and integrin-binding factors are upregulated [18]. Chronic low-grade inflammation is known to play a critical role in the pathogenesis and progression of AD. Increased BBB permeability contributes to elevated leukocyte infiltration, particularly neutrophils, and thereby mediates vascular inflammation in the brain. Leukocytes attach to cerebral endothelial cells and migrate to the brain parenchyma, particularly in the hippocampus and other limbic structures [53,54]. Leukocyte infiltration is a multistep process that is mediated by adhesion molecules such as selectins, integrins, and the immunoglobulin superfamily [55]. The α4β1 integrin, also known as CD49d/CD29 or very late antigen-4 (VLA-4), is the most predominant β1 integrin expressed on leukocytes [56] and also plays an essential role in T cell trafficking during various inflammatory responses [57], as well as in CNS pathologies such as multiple sclerosis and experimental autoimmune encephalomyelitis [55,58]. Pietronigro et al. demonstrated that the α4β1 integrin is a pivotal mediator of leukocyte adhesion on activated endothelial cells and blocking the α4 chain with specific antibody inhibits rolling interactions in cortical venules in 3xtg-AD mice, indicating that VLA-4 promotes the leukocyte–vascular interactions in AD mice [56]. They found an age-dependent increase in the proportion of α4-integrin-expressing CD4+ cells in 3xTg-AD mice and an inhibition of α4β1 integrin improved cognitive function as evidenced by improved performance in a Y maze, contextual fear conditioning, and MWM tests [56]. Taken together, the study shows that the therapeutic potential of α4β1 integrin inhibition interferes with disease progression and cognitive impairment. Further, α4 integrins blockage attenuated leukocyte–endothelial interactions and thereby significantly inhibited neuropathological hallmarks such as Aβ deposition and tau hyperphosphorylation.

From the foregoing, integrins may be a worthwhile research focus as a therapeutic target in cognitive impairment and dementia.

## 3. Improving the Translational Perspective for Modulating Integrin Signaling in the Context of Cognitive Impairment

A number of potential therapeutic integrin targets are currently under investigation or have been shown previously to ameliorate the impact of cognitive impairment in the context of the two leading types of dementia. We will outline some of the findings (Table 1) and briefly indicate potential areas of growth in this area. C16 (KAFDITYVRLKF), a selective peptide inhibitor for αvβ3, was shown to interfere with the transmigration of leukocytes and inflammation [59]. Moreover, C16 has shown a beneficial effect on the ALS/Parkinsonism dementia complex (PDC), representing symptoms analogous to AD’s such as dementia and Parkinsonism, when administrated along with angiopoietin 1, a nerve growth factor. A combination treatment of C16 with angiopoietin 1 improved oxidative stress, neuroinflammation, and cognitive function in a rat model of PDC induced by Beta-N-methylamino-L-alanine (L-BMAA) [60]. In addition to leukocytes, platelets also play a critical role in the development and progression of VCID and AD as they harbor amyloid precursor protein (APP) and secretases required to cleave the APP [61], and aberrant platelet activation has been reported in AD patients [62]. Lee et al. demonstrated that Aβ1–40 stimulated aberrant reactive oxygen species (ROS) production in human platelets and the activation of integrin αIIbβ3 through a PKC-δ-dependent mechanism [63]. Treatment with Rosmarinic acid, a phyto-polyphenolic compound, attenuated platelet adhesion through the modulation of ROS production and inhibition of αIIbβ3 signaling.

Recently, Ortiz-Sanz et al. demonstrated the therapeutic potential of the N-terminal signal peptide of β1 integrin localized at the first 20 amino acids, towards AD. The β1 integrin binds to Aβ oligomers and attenuated ROS generation in primary astrocyte cultures treated with Aβ oligomers [64]. Further, intrahippocampal administration of recombinant integrin β1 signal peptide prevented both astrogliosis and microgliosis and endoplasmic reticulum stress mediated by Aβ oligomers in vivo.

We have previously reported that the domain V (DV) 85-kDa protein fragment of the extracellular matrix proteogylcan perlecan is generated by proteolysis and could modulate α2β1 signaling induced by Aβ in vitro [65]. Perlecan DV is an α2 integrin ligand shown to inhibit Aβ-induced neurotoxicity in human cortical neurons in vitro through the α2β1 integrin receptor and a p-c-jun-dependent mechanism [66]. Aβ is a ligand for both α2β1 and αvβ1 confirming their role in AD pathology [67]. Further, DV (and its 25-kDa subfragment, LG3) administration has blocked Aβ toxicity in mouse fetal hippocampal neurons through the inhibition of c-Jun and caspase-3 [64] demonstrating the therapeutic potential of perlecan subunits.

Although the above stated studies have heralded the current thinking that integrins have a strategic role towards improving brain health and ameliorating dementia-like effects, the majority of preclinical studies continue to pursue translational relevance whilst not considering important factors associated with dementia [68,69,70,71]. For example, given that VCID and AD are diseases of advance age and affect both males and females, more work lies ahead in the use of appropriately aged models as well as the careful consideration of sex as a biological factor. Moreover, while we recognize that there exists no perfect model for translational studies, VCID more often results from hypertension and other comorbidities such as metabolic syndrome and diabetes mellitus [72,73,74]. Hence, the above translationally relevant factors ought to be considered for future studies seeking to develop therapeutic strategies for dementia whilst focusing on the roles of integrins in memory impairments.

**Table 1:** Summary of studies on modulation of integrin signaling in AD/dementia.

Disease Model	Inhibitor/Modulator	Inference	Reference
3xTg-AD mice	500 μg of the α4-integrin-specific antibody	Attenuated neuropathological hallmarks of AD, such as microgliosis, Aβ load, and tau hyperphosphorylation. The α4 integrin blocking attenuated leukocyte trafficking and improved cognitive impairment and AD neuropathology	Pietronigro et al., 2019 [56]
C57BL6/J mice with Intrahippocampal Aβ oligomers injection	recombinant integrin β1 N-terminal signal peptide	Inhibited Aβ-induced ROS generation in primary astrocytes Inhibited astrogliosis and ER stress in mouse of AD	Ortiz-Sanz et al., 2022 [64]
Rat model of ALS/PDC model (induced by L-BMAA)	C16 peptide KAFDITYVRLKF along with angiopetin 1 (Ang1)	Attenuated oxidative stress and inflammatory response Improved cognitive and motor function	Cai et al., 2018 [60]
Human and mouse cortical neurons treated with Aβ	Domain V and LG3 of perlecan	Inhibited Aβ-induced neurotoxicity in an α2 integrin and c-Jun dependent manner	Wright et al., 2010 [67]
Mouse hippocampal neurons treated with Aβ_42_	DV and LG3	DV and LG3 inhibited the α2β1 integrin receptor and prevented Aβ from binding	Parham et al., 2016 [65]
Human platelets treated with Aβ1–40	Rosmarinic acid	Aβ1–40-induced platelet adhesion is ameliorated by RA through the inhibition of NADPH oxidase/ROS/PKC-δ/integrin αIIbβ3 signaling pathways	Lee et al., 2021 [63]

## 4. Conclusions

From the foregoing, the role of integrins in memory function and retention stems from their involvement in the formation and function of the dendritic spine. Several studies have shown that the targeted integrin inhibition of α3, α5, or β1 leads to a decrease in dendritic size and quantity, as well as LTP. Findings from multiple behavioral tests have also shown that the inhibition of these same integrins results in mice with impaired memory. Hence, β1, α5, and α3 integrins functioning at the synapse are crucial for proper memory function. However, it is still unclear if the observed deleterious effects on memory are a direct cause of integrin attenuation or due to the loss of communication between cells. Increasing research in this area could uncover if different integrins play different roles in memory formation and retention, as well as determine if there are distinct pathways for the creation of new memories and their recall.

Integrins and BBB disruption have both been linked with age-associated memory deficits. The disruption of α5 integrins mediating angiogenesis during the acute phase of vascular supply interruptions to the brain disrupts the integrity of the BBB and may be implicated in cognitive dysfunction. There is, currently, a lack of direct evidence on the specific roles of integrins in models of VCID and more studies are warranted to consider this direction given the global prevalence of VCID. Although reports have linked αVβ3, α4β1, and αLβ2 integrins to the pathology of AD, little is known of the role of these integrins in models with age-related comorbidities or in female models.

To better translate experimental findings, especially in the area of potential therapy that aims to target integrins, efforts must be intensified in modeling human disease conditions as well as in the inclusion of sex as a biological factor. Since experimental models are insufficient and to expand the true translational potential of bed-bench collaboration, more efforts should be directed towards integrin analyses of post-mortem brain tissues from individuals who were diagnosed with VCID and AD. One major step towards this endeavor will be to establish a subregional hippocampus tissue biobank and registry, which will preserve historical patient records as well as hippocampal brain specimens. Clearly, integrins are associated with memory function in VCID and AD pathobiology/models and an increased understanding of their specific roles and therapeutic potentials in ameliorating cognitive deficits is urgently warranted.
